# Difficulties of caregivers of cow's milk allergy patients in understanding the labeling of processed products

**DOI:** 10.1186/1939-4551-8-S1-A62

**Published:** 2015-04-08

**Authors:** Marina Amoroso, Mariana Forti, Cleonir Beck, Glauce Yonamine, Andrea Gushken, Mayra Dorna, Ana Paula Moschione Castro, Antonio Carlos Pastorino, Cristina Jacob

**Affiliations:** 1 Unit of Allergy and Immunology, Brazil; 2 Nutritionist of Division of Nutrition, Brazil

## Background

Cow's Milk Allergy (CMA) is the main food allergy (FA) in children and its treatment includes restricted milk diet. The correct identification of terms that mean milk ensures the efficacy of treatment.The aim of this study was to identify the main factors involved in the caregivers’ ability to recognize the presence of milk in products labels.

## Methods

Cross-sectional and descriptive study was carried out with CMA patient’s caregivers in follow-up at a pediatric reference center for FA. All of them were previously instructed about labels. This study included a questionnaire about the knowledge regarding the labels reading. Caregivers evaluated 20 labels (15 of foods, 3 of medicines and 2 of cosmetics) and should decide if the product was safe and the reason it can or not be offered to the patients. 15/20 contained words meaning milk protein. Results were expressed in number of labels reading (20 labels/caregivers)

## Results

Twenty-eight caregivers fulfilled the questionnaire. The caregivers were 78.5% mothers and 21.5% fathers and about their schooling years 78.2% finished high school or college degree. The median of patient’s follow-up was 12 months (0.03-144). Twenty caregivers deal with patients that presented at least one episode of anaphylaxis. All caregivers referred label reading: 25 read every time they buy the product and only 9 read the labels after buying, before storing and offering.Labels were correctly read in 75.7 % being lactose, casein and whey protein the most common correctly identified terms. The most common mistakes were related to the terms lactil and caramel color. About medicines the most difficulties was to localize de terms related to milk in the package leaflet. Main factors regarding wrong comprehension were small or unclear printing, difficult localization and ingredients in foreign language

## Conclusions

Labels reading is part of food allergy treatment and demands constant reinforcements stressing the necessity of continuous label reading. Labels quality must improve in many aspects in order to help caregivers understanding of milk labels and avoid patient’s accidental intake.

